# Virtual Reality-Based Center of Mass-Assisted Personalized Balance Training System

**DOI:** 10.3389/fbioe.2017.00085

**Published:** 2018-01-12

**Authors:** Deepesh Kumar, Alejandro González, Abhijit Das, Anirban Dutta, Philippe Fraisse, Mitsuhiro Hayashibe, Uttama Lahiri

**Affiliations:** ^1^Department of Electrical Engineering, Indian Institute of Technology Gandhinagar, Gandhinagar, India; ^2^INRIA Camin team and LIRMM, University of Montpellier, Montpellier, France; ^3^Conacyt-Universidad Autonoma de San Luis Potosi, San Luis Potosi, Mexico; ^4^AMRI Institute of Neuroscience, Kolkata, India; ^5^Department of Biomedical Engineering, University at Buffalo, Buffalo, NY, United States; ^6^Department of Robotics, Tohoku University, Sendai, Japan

**Keywords:** ankle strategy, balance rehabilitation, center of mass, Kinect, stroke, virtual reality

## Abstract

Poststroke hemiplegic patients often show altered weight distribution with balance disorders, increasing their risk of fall. Conventional balance training, though powerful, suffers from scarcity of trained therapists, frequent visits to clinics to get therapy, one-on-one therapy sessions, and monotony of repetitive exercise tasks. Thus, technology-assisted balance rehabilitation can be an alternative solution. Here, we chose virtual reality as a technology-based platform to develop motivating balance tasks. This platform was augmented with off-the-shelf available sensors such as Nintendo Wii balance board and Kinect to estimate one’s center of mass (CoM). The virtual reality-based CoM-assisted balance tasks (Virtual CoMBaT) was designed to be adaptive to one’s individualized weight-shifting capability quantified through CoM displacement. Participants were asked to interact with Virtual CoMBaT that offered tasks of varying challenge levels while adhering to ankle strategy for weight shifting. To facilitate the patients to use ankle strategy during weight-shifting, we designed a heel lift detection module. A usability study was carried out with 12 hemiplegic patients. Results indicate the potential of our system to contribute to improving one’s overall performance in balance-related tasks belonging to different difficulty levels.

## Introduction

Balance disorder is a common problem in majority of poststroke survivors, and it can have a significant impact on one’s functional independence in day-to-day life (Stroke, 2012). Persons with hemiplegia and balance impairments often show altered weight distribution patterns, such as, healthy side bears most of the body weight compared to weaker side of the body while performing activities (Pereira et al., 2010). Also, they exhibit smaller excursions on the weaker side of their body when shifting their weight around the base of support (Tyson et al., 2006). These problems result in loss of static and dynamic stability and restrict the mobility of stroke survivors (Hamzat and Fashoyin, 2007) with increased risk of falls (Lamb et al., 2003). The incidence of falls has been reported to be up to 73% in the first year poststroke (Verheyden et al., 2013).

Current clinical practice for balance training includes exercises directed by trained clinicians. Although conventional treatments are effective in improving balance, the limitations such as scarcity of adequately trained healthcare professionals (Yatar and Yildirim, 2015), high cost of repetitive one-on-one services in specialized health clinics (Bateni, 2012), requirement to commute to distant health centers to avail rehabilitation services, etc. may lead to compromised treatment effects. Thus, technology-assisted balance rehabilitation approaches can provide an accessible, quantifiable, and individualized alternative.

Among the alternative technology-assisted platforms, we chose virtual reality (VR)-based systems since these can offer an individualized, cost-effective, safe, interactive, and repetitive practice environment with variations (Darekar et al., 2015) that are often motivating (Gil-Gómez et al., 2011) for the participants. Various researchers have already shown the potential of VR-based balance rehabilitation to contribute to balance recovery compared with that by conventional therapy in individuals with chronic stroke (Darekar et al., 2015; Lloréns et al., 2015). Most of the existing VR-based systems addressing balance issues have used off-the-shelf games (designed with an entertainment perspective) to rehabilitation (Gil-Gómez et al., 2011; Cho et al., 2012; Rajaratnam et al., 2013; Yatar and Yildirim, 2015). Also, the balance tasks offered to the participants by these studies are not individualized, that is not adaptive to one’s performance which is a critical requirement for effective rehabilitation (Choi et al., 2011; O’Sullivan and Schmitz, 2016). Given the inherent advantages of VR such as, flexibility in design, controllability, malleability, etc. (Burdea, 2003), designing VR-based balance tasks that can quantify one’s performance in a task in terms of weight-shifting capability, be adaptive to one’s performance and offer real-time feedback on one’s weight shifting is feasible.

For offering real-time feedback during a balance task, previous VR-based research studies using off-the-shelf games (Barclay-Goddard et al., 2004; Sayenko et al., 2010; Gil-Gómez et al., 2011; Cho et al., 2012; Rajaratnam et al., 2013; Yatar and Yildirim, 2015) have used one’s center of pressure (CoP) measured by Wii balance board (WiiBB; Nintendo Co., Ltd., Kyoto, Japan) instead of the center of mass (CoM). The use of CoP in the context of geriatric assessment or clinical settings may be preferable over CoM given easier estimation of one’s CoP (Pasma et al., 2014) along with lesser challenge posed by CoP-based tasks. However, research studies report that to ensure that the balance task is sufficiently challenging, the CoM-based approach can be preferred over the CoP-based approach (Lizama et al., 2015). Measurement of one’s CoM during a dynamic balance task might be cumbersome, space intensive and costly due to the usage of marker-based motion capture systems used presently (Lizama et al., 2014). However, in contrast, one can use marker-less motion capture systems (González et al., 2014, Hayashibe et al., 2017) such as Kinect to measure one’s CoM.

In our present study, we have designed a virtual reality-based CoM-assisted balance task (Virtual CoMBaT) platform where the participants are asked to interact with VR-based balance task by shifting weight while standing. To estimate one’s personalized CoM, we have used the statistically equivalent serial chain (SESC) method comprising of Identification and Measurement stages as proposed by González et al. (2014) and Hayashibe et al. (2017). The Identification stage uses inexpensive off-the-shelf sensors namely Kinect and WiiBB to identify SESC parameters to estimate one’s body mass distribution. Subsequently, the Measurement stage computes one’s personalized CoM using the Kinect sensor and the SESC model parameters while the participants performed the balance tasks. During the balance tasks, the participants were asked to follow ankle strategy. In fact, among the three main postural strategies, namely, ankle, hip, and step strategies (Lee et al., 2015), the ankle strategy enabling muscle contraction of the ankle joint is most commonly used for addressing balance-related issues (Lee et al., 2014). To assist the participants in following ankle strategy during the balance task, we designed a heel lift detection (HLD) module interfaced wirelessly with the VR-based task platform.

The objectives of our present study are threefold: (i) develop a CoM-assisted balance training platform having a VR-based system (Virtual CoMBaT) augmented with Kinect to offer balance rehabilitation exercises in an individualized and adaptive manner based on one’s performance capabilities and (ii) develop a HLD module to assist in maintaining ankle strategy (iii) conduct a usability study with the Virtual CoMBaT system, with an aim to understand the users’ perspective on the usage of the system and implications of such a system on task performance of individuals having balance disorders. Additionally, we plan to use one’s performance indicators derived from the usability study with the Virtual CoMBaT system as possible metrices to quantify participants’ initial (residual) weight shifting ability.

This article is organized as follows: Section “Materials and Methods” presents the materials and the methodology used, Section “Result” discusses the results obtained in the usability study. Finally, Section “Discussion and Limitation” summarizes the research findings and discusses the limitations of the current study as well as the direction of future research.

## Materials and Methods

### VR-Based CoM-Assisted Balance Training (Virtual CoMBaT) System

The Virtual CoMBaT system consisted of five modules, namely, (A) personalized CoM estimation, (B) design and control of VR-based balance rehabilitation tasks, (C) monitoring of ankle strategy, (D) performance evaluation, and (E) task switching modules.

#### Personalized CoM Estimation

**Figure 1:**
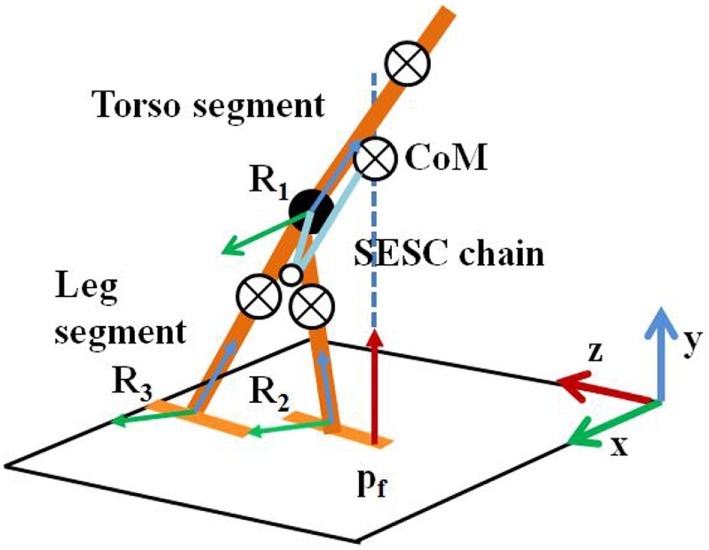
Multisegment skeleton model. SESC, statistically equivalent serial chain; CoM, center of mass.

Personalized CoM was estimated using SESC method proposed by Espiau and Boulic (1998). This method translates one’s mass distribution to the link-length of a linked chain model. These links are subsequently used to compute one’s CoM position. Cotton et al. (2008, 2009) have demonstrated the use of a multilink SESC chain constrained to a plane for estimating one’s CoM position. The SESC procedure used for personalized CoM estimation has been validated for an elderly population with no balance issues (Cotton et al., 2011) and for young, healthy subjects (González et al., 2015, Hayashibe et al., 2017). Using the SESC method, the CoM of an articulated structure can be computed, after attaching a frame (*R_i_*) to each link, using Eq. 1 (González et al., 2014).

(1) $$c=\left[E\;A_{1}\;A_{2}\ldots A_{n}\right]{\left[p_{0}\;r_{1}\;r_{2}\ldots r_{n}\right]}^{T},$$

where *c* is CoM of an articulated structure, *E* is an identity matrix, *A_i_* is a 3 × 3 matrix describing the orientation of a link, and *p*_0_ is the position of the origin of *R*_1_ (a floating frame attached to the torso of skeleton as a base for the SESC) with respect to the global reference frame (Figure 1). The values of *r_i_* can be explicitly determined as a function of the linked masses and geometries. The subject-specific *r_i_* values were identified experimentally during SESC parameter Identification stage. The number of links/segments considered for the model should be sufficient to accurately describe the performed motion. In the work presented by González et al. (2014, 2015), the Identification stage used a nine link SESC model with 40 static postures that consisted of squatting, standing on one leg and doing different static postures, etc. However, this work involved healthy participants. Again, none of these participants had balance disorders that might pose difficulty or make it infeasible to do 40 static postures while standing on a WiiBB. Since our participants were poststroke individuals with balance disorders, we tried to carry out an Identification stage that needed fewer static postures (for details, see Experimental Protocol). For this, in our present study, we optimized the SESC algorithm by modeling the participant’s motion using three segments (one torso and two legs) as shown in Figure 1. The participants were not asked to perform any squatting task such as, knee bending. Instead they were asked to make only three static postures, namely, lean forward and lift two legs, one at a time away from the body.

The SESC method maps the CoP measure (from WiiBB) to the CoM (from Kinect) ground projection during static postures in the Identification stage (González et al., 2014, González et al., 2015). By using the optimized SESC algorithm, the difference between the measured CoP (from WiiBB) and estimated CoM was approximately 5% along mediolateral and 12% along the anteroposterior directions on an average, that are comparable with the observations made in the previous studies (González et al., 2014). In order to minimize the effect of the difference between the measured CoP and estimated CoM as found during the Identification stage, for computing one’s task performance score during the Task Execution stage (see Experimental Protocol) using the value of the CoM, we considered the relative change in the CoM position in an individualized manner.

#### Design and Control of VR-based Balance Rehabilitation Tasks

**Figure 2:**
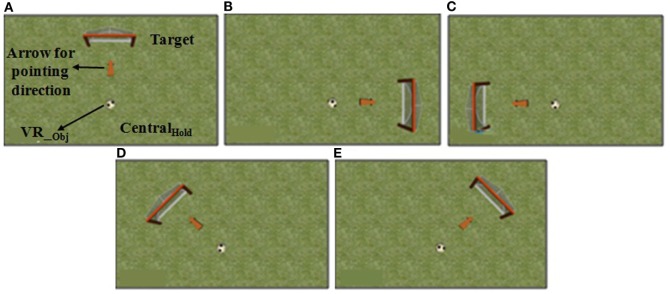
Graphical user interface of a virtual reality (VR)-based balance training task in **(A)** North, **(B)** East, **(C)** West, **(D)** North-West, and **(E)** North-East directions.VR__obj_ = virtual object.

**Figure 3:**
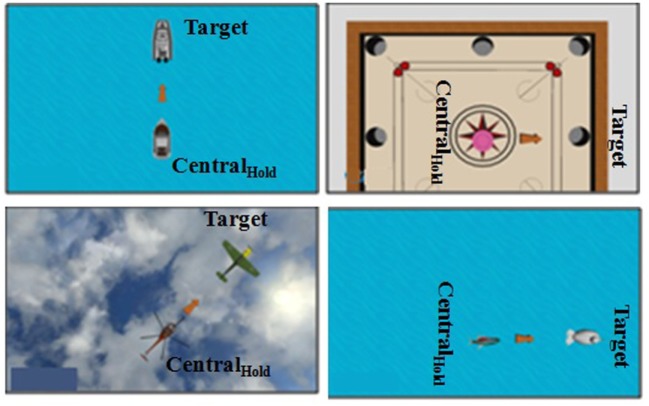
Graphical user interface of few virtual reality (VR)-based balance training tasks.

In this study, the VR-based tasks were developed to (i) leverage one’s weight shifting ability in different directions while following ankle strategy and (ii) quantitatively estimate one’s balance capability during the weight-shifting task. We used Vizard software toolkit (from Worldviz Llc.) to design VR environments. We created a database of 30 unique combinations of VR-based environments (such as, road, playground, river, etc.) and virtual objects (such as, car, ball, fish, etc.) related to stimuli that one often might come across in daily living and entertainment. The tasks required participants to shift their weight (by varying CoM position) in predefined directions, namely, North (*N*), East (*E*), West (*W*), North East (*NE*), and North West (*NW*) while maintaining stability that is moving within their limit of stability by following ankle strategy (without lifting their heel from the ground surface). We did not consider the South (*S*) direction since, most of our participants were over-weight in their physique. While standing upright on the WiiBB, the CoP were displaced toward the *S* direction. Thus, usage of *S* direction for a task would have most likely added a confounding factor to the task performance score. So, we did not consider the *S* direction for our VR-based tasks. The participants were asked to maneuver the virtual objects (*VR_obj_* henceforth) in the VR environment from their initial (*Central_Hold_*) to target (*Target*) positions (Figures 2 and 3) by shifting weight while displacing their CoM. Figures 2 and 3 (similar to those in our companion article Verma et al., 2017) show example of the Graphical user interface of VR-based tasks used. Our tasks were designed for two difficulty levels (DL1 and DL2) with weight-shifting thresholds being individualized.

##### Estimation of Individualized Threshold for VR-Based Task

In our present work, we wanted to individualize the system based on one’s ability to shift weight in different directions. For this, we determined individualized thresholds as far as weight-shifting ability is concerned. Before starting the study, we asked the participant to stand on the ground and shift weight forward [North (*N*)], toward left [West (*W*)], and right [East (*E*)] as much as possible while ensuring that ankle strategy was followed. Simultaneously, we recorded the participant’s corresponding CoM position using SESC method to estimate the individualized threshold capability of weight-shifting. The direction-specific thresholds were decided from the maximum CoM displacement (ΔCoM_max_) a participant could achieve in the *N*, *E*, and *W* directions. The ΔCoM_max_ for each direction was chosen as the minimum required CoM displacement for reaching the *Target* (Figures 2 and 3) position for the DL2 task and 80% of ΔCoM_max_ for DL1 task. Thus, DL2 tasks required one to shift weight more than that for DL1 tasks. The threshold for DL1 tasks was chosen to be 80% of ΔCoM_max_ as an initial approximation. In fact, this value can be changed based on the study design.

##### CoM-VR Integration

Virtual CoMBaT recorded the CoM trajectories along *N, E, W, NE*, and *NW* directions corresponding to one’s weight-shifting during the VR-based Tasks. The CoM positions were mapped to a Virtual object (*VR_obj_*) in real-time using Eq. 2.

(2) $${\left[\begin{array}{c} x\\ y \end{array}\right]}_{\text{V}{\text{R}}_{\text{obj}}}=\left[\begin{array}{cc} {\text{CoM}}_{x} & \text{0}\\ \text{0} & {\text{CoM}}_{y} \end{array}\right]\left[\begin{array}{c} \in_{\text{1}}\\ \in_{\text{2}} \end{array}\right],$$

where ∈_1_ and ∈_2_ are scaling constants for the VR_Obj_ coordinates (*x*, *y*) corresponding to the CoM position (CoM*_x_*, CoM*_y_*) on the computer monitor.

#### Monitoring of Ankle Strategy during Task Execution

**Figure 4:**
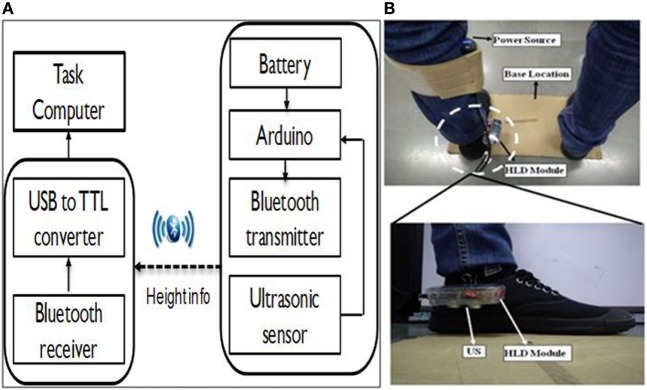
**(A)** Block diagram and **(B)** placement of HLD module on the shoe.

In our present study, we wanted to ensure that the participants followed ankle strategy which is considered as important in standing balance task (Lee et al., 2014). While doing weight-shifting task by adhering to ankle strategy, one’s body acts as a single segment pendulum centered about the ankle joint (Hwang et al., 2009). In order to ensure that the ankle strategy is followed, the participants are expected to not lift their heel from the ground while shifting their weight in different directions. Thus, we designed a shoe-based HLD module that wirelessly communicated with our VR-based system to (i) provide an audio alarm as a feedback to the participant and (ii) add a penalty factor to the participant’s performance score in case the ankle strategy was not followed. The HLD module consisted of an ultrasonic sensor (US), an Arduino board, a Bluetooth transmitter and receiver pair and a universal serial bus to TTL transistor–transistor logic converter. Figure 4A shows the block diagram of HLD module and Figure 4B shows the placement of HLD module on the shoes worn on the affected leg. The HLD module was attached to the medial side of the shoe closer to the inner ankle bone of the affected leg with the US facing downwards toward the ground. A participant was asked to stand upright with his heels touching the ground surface. Then the US sensor mounted on the participant’s shoe was used to measure the initial height (*d*_ini_ in mm) between US sensor facing ground and from the ground surface. This height changes when one lifts his heel above the ground during the task execution. The output from the US sensor was processed by the microcontroller of the Arduino board that was transmitted wirelessly to the task computer to detect the heel lift by using Eq. 3.

(3) $$\text{Ankle Strategy}=\left\{\begin{array}{cc} \text{Followed;} & \text{if}\;d_{\text{ins}}<d_{\text{Limit}}\\ \text{Not Followed;} & \text{if}\;d_{\text{ins}}\geq d_{\text{Limit}} \end{array}\right.,$$

(4) $$d_{\text{Limit}}=d_{\text{ini}}+d_{\text{th}},$$

where *d*_ins_ = instantaneous height of the heel above the ground as measured by the US and *d*_th_ = 20 mm = height tolerance for HLD. In our study, we have chosen one’s threshold height for HLD (*d*_th_) as 2 cm as a typical case while considering our participant pool. Our participants with balance disorder often demonstrated small movement in different directions while standing in order to stabilize their standing posture within the tasks. These small movements often resulted in variation of *d*_ins_ that led to false alarm related to ankle strategy being not followed (inferring false trigger of the HLD module). Again, our participants being poststroke patients, often demonstrated foot inversion while standing. Since our HLD module was attached to the inner medial side of the paretic limb, we wanted to make sure that the foot inversion while standing do not lead to false triggers of the HLD module. The aim of using the HLD module was to sense any possible heel lift only during one’s weight shifting so as to adhere to ankle strategy. Also, we did not provide any tolerance or margin between the two states of ankle strategy, namely, Followed or Not Followed (Eq. 3). The HLD module transmitted *d*_ins_ (for the heel lift information) in real-time to the serial port of task computer presenting the VR-based tasks, wirelessly. The frame rate of transmission of the HLD information was chosen to be 60 Hz.

Based on the *d*_ins_ value, if the ankle strategy was Not Followed then a penalty factor was added in the performance score (described below). Otherwise, no penalty factor was considered in the performance evaluation.

#### Performance Evaluation Module

We wanted to evaluate and understand a participant’s ability to shift weight in different directions while interacting with the VR-based tasks presented by the Virtual CoMBaT system. The Virtual CoMBaT system computed the performance scores for the tasks of DL1 and DL2 in each of the five directions based on (i) *P*_S1_: length of trajectory (T_L_) of one’s CoM before reaching the Target position, (ii) *P*_S2_: deviation of one’s CoM from the instructed straight path between Central_Hold_ and Target positions, (iii) *P*_S3_: measure of one’s ability to hold his weight at the Target position for hold time (*H*_T_) of 1 s, and (iv) *P*_S4_: penalizing factor to discourage one’s heel lifting (Verma et al., 2017).

**Figure 5:**
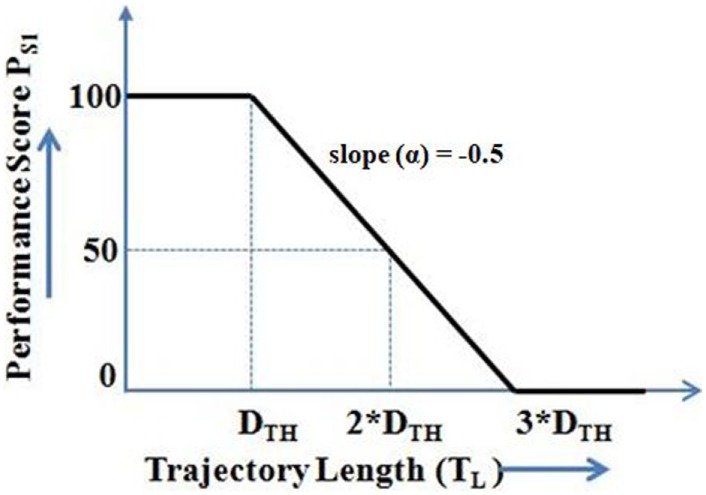
Evaluation of the participant’s CoM trajectory for body sway while shifting weight (*P*_S1_).

The first metric (*P*_S1_) evaluated the participant’s CoM trajectory for body sway while shifting weight by using Eq. 5.

(5) $$P_{\text{S1}}=\left\{\begin{array}{cc} 100; & \text{if}\;T_{\text{L}}\leq D_{\text{TH}}\\ 100-\propto \times \left(\frac{T_{\text{L}}-D_{\text{TH}}}{D_{\text{TH}}}\right)\times 100; & \text{if}\;D_{\text{TH}}<T_{L}<3\times D_{\text{TH}},\\ 0; & T_{\text{L}}\geq 3\times D_{\text{TH}} \end{array}\right.$$

(6) $$D_{\text{TH}}=1.8\times T_{\text{L}},$$

where *T*_L_ is the length of the participant’s CoM trajectory between the Central_Hold_ and Target positions in each direction. In Eq 5, *P*_S1_ can have three possible values depending on *T*_L_. For values of *T*_L_ < *D*_TH_ and *T*_L_ ≥ 3 × *D*_TH_, *P*_S1_ was programmed to be scored as 100 and 0, respectively. For the intermediate values of *T*_L_, we used a multiplication factor of α = 0.5 so as to linearize the penalty factor due to increase in the value of *T*_L_ between *D*_TH_ and 3 × *D*_TH_ (Figure 5). The range of the values of *T*_L_ (as function of *D*_TH_) was chosen as an initial approximation. This can be changed based on the study design.

The value of *D*_TH_ was decided based on a pilot study with healthy participants [*n* = 7; mean (SD) = 39 (16.45) years]. In this pilot study, we computed the length of one’s CoM trajectory to reach the Target position in different directions. Then the value of *D*_TH_ was computed by averaging the distance traveled by the participants’ CoM while shifting weight in different directions. It was found to be 1.8 × the length of the straight line path between the Central_Hold_ and Target positions (Eq. 6).

The second metric (*P*_S2_) evaluated the quality of participant’s weight shift [in terms of deviation (*D*_A_)] from the instructed direction (defined by θ*_x_* = 0° for *East*; 45° for *North-East*; 90° for *North*; 135° for *North-West*; and 180° for *West*) with a tolerance range (θ_RANGE_) of ±22.5° around the instructed direction.

(7) $$P_{\text{S2}}=100-\propto \times \left(\frac{\text{abs}\left(\theta_{x}-D_{\text{A}}\right)}{\theta_{\text{RANGE}}}\right)\times 100,$$

Here, α = 1/2.

The third metric (*P*_S3_) was considered to encourage stable weight-shifting by the participant.

(8) $$P_{\text{S3}}=\left\{\begin{array}{cc} 100; & \text{if}\;H_{\text{T}}\geq 1s\\ 0; & \text{if}\;H_{\text{T}}<1s \end{array}\right.,$$

The fourth metric (*P*_S4_) was used to penalize the participant for heel lift during weight-shifting.

(9) $$P_{\text{S4}}=\left\{\begin{array}{cc} -100; & \text{Ankle Strategy}\prime \text{NotFollowed}\prime \\ 0; & \text{Ankle Strategy}\prime \text{Followed}\prime \end{array}\right.,$$

Therefore, the weighted performance score $\left(P_{\text{s}}^{x}\right)$ for each of the five directions (*x* = *East/North East/North/North West/West*) was calculated as:

(10) $$P_{\text{s}}^{x}=0.5P_{\text{s1}}^{x}+0.25P_{\text{s2}}^{x}+0.25P_{\text{s3}}^{x}+0.2P_{\text{s4}}^{x},$$

As suggested by the therapist, we assigned a higher weightage to *P*_s1_ (0.5) than *P*_s2_ (0.25) and *P*_s3_ (0.25). For improved balance ability during weight-shifting, reduced postural sway (indicated by *P*_s1_) is often more important than remaining close to the *Target* location (*P*_S2_) and holding the shifted weight (*P*_S3_) and it was realized in our study through higher weightage being assigned to *P*_s1_. Also, a penalty factor (0.2) was used to discourage a participant from lifting his heel while shifting weight. The penalty factor of 0.2 was considered as an initial approximation that can be modified in future based on study design.

The final performance score (*P_s_*) for each task was computed from the average of the performance scores for all the five directions by using Eq. 9.

(11) $$P_{\text{s}}=\frac{1}{5}\sum_{x}P_{\text{s}}^{x}.$$

Again, our participants were hemiplegic. Thus, we wanted to quantify one’s residual weight-shifting ability for each direction (*N*, *E*, *W*, *NE*, and *NW*) when they came in for the study. For this, we calculated one’s normalized equivalent performance (NEP) based on their performance score in the *First Attempt* of DL1 and DL2 by using Eq. 12.

(12) $$\text{NEP}=\left(\frac{1}{3}\times P_{{\text{f}}_{\text{DL1}}}+\frac{2}{3}\times P_{{\text{f}}_{\text{DL2}}}\right)/100.$$

While the participants interacted with many task trials at each difficulty level (DL1 and DL2), $P_{{\text{f}}_{\text{DL1}}}$ and $P_{{\text{f}}_{\text{DL2}}}$ were their individual performance scores for each of the five directions (*N, E, W, NE*, and *NW*) in the *First Attempt* of DL1 and DL2 tasks, respectively. The NEP score was used, since, the tasks belonging to DL1 and DL2 were of different challenge levels. The DL2 tasks required larger weight-shifting than DL1 tasks for task completion. Thus, we estimated NEP as a weighted average of the scores in the two subtasks.

#### Task Switching Module

**Figure 6:**
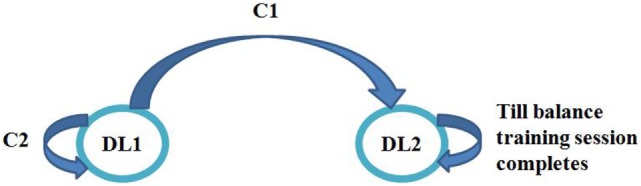
State machine representation for task switching.

The Virtual CoMBaT system switched tasks presented to the participants while being adaptive to their individualized performance score in a task. The task switching algorithm had two switching conditions (C1 and C2; Table 1). We were interested to see one’s trajectory of improvement in performance score in each difficulty level. If a participant was interacting with a task of DL1, the Virtual CoMBaT looked for whether there was (i) improvement [(Pf [CT] − Pf [PT]) > 0; *P*_f_ = Percentage performance score, CT = current task, and PT = previous task] in his/her task performance through repeated exposure to tasks of DL1 (Table 1 and Figure 6) and (ii) his/her performance in any of the task trials of DL1 was “Adequate” (Condition “C1”). Again, even after performing adequately in one of the task trials of DL1, it might so happen that due to repeated exposures to the tasks of same challenge level (DL1), the participant might lose interest. In that case, to regain back the interest, the Virtual CoMBaT offered task of higher challenge level (DL2), given that this difficulty level was decided based on their maximum weight-shifting capability before participating in the study (see Experimental Protocol). On reaching DL2, the participant will continue at that level until the task completion time (20 min in our case). In our case, if a participant’s performance score was ≥70%, then his performance was considered as “Adequate,” else, it was considered as “Inadequate.” We have chosen the performance threshold score of 70% for “Adequate” since, literature indicates 70% as the average initial exercise performance for rehabilitation tasks (Metzger et al., 2014), for outpatient clinics (Jack et al., 2010) and technology-assisted skill learning (Young et al., 2014).

**Table 1:** Task switching criteria.

Condition	Description	Action
C1	*P*_f_ (CT)*_i_*_+1_ < *P*_f_ (PT)*_i_* and *P*_f_ (CT)*_i_* > 70%; where *i* = 1, …, *n*	Move to higher difficulty level (except for DL2)
C2	*P*_f_ (CT)*_i_*_+1_ ≥ *P*_f_ (PT)*_i_*, where *i* = 1, …, *n*	Remain in same difficulty level

#### System Usability-related Questionnaire

In order to understand the usability of the Virtual CoMBaT system, a five point likert scale (Likert, 1932) questionnaire was used to get the participant’s feedback. We framed five questions in order to understand users’ views on their usage of our Virtual CoMBaT system. For this, we chose few questions (as were relevant for our study) from the User Suitability Evaluation Questionnaires used by Gil-Gómez et al. (2013). From this Questionnaire, we chose three questions. The first question was “Did you face any difficulty in understanding the task?” (Q1). The idea was to understand whether the information provided by Virtual CoMBaT system during the task was clear. The second question was “Did you find the task interesting?” (Q2). This was asked so as know how they felt in interacting with our system. Again, although our study needed participation for one day, yet, since this system was designed with rehabilitation in mind that might need its usage over extended period, we asked them the third question, namely, “Do you think that the usage of this system would be beneficial to you?” (Q3). In addition, we wanted to understand whether the use of our Virtual CoMBaT system was motivating to the participants. For this, we asked them two more questions, namely, “Will you agree to interact with our system again?” (Q4) and “Will you refer others to participate in our study?” (Q5).

### Participants

The study was carried out at local physiotherapy clinics, and we followed institutional research ethics. In the present usability study, 12 stroke survivors (S1–S12) [mean (SD) = 55.25 years (10.34)] were enrolled from the physiotherapy clinics where they were undergoing therapy. Table 2 shows the participants’ characteristics. The inclusion criteria were (1) ability to follow the instructions and (2) ability to stand and shift weight without orthopedic aids.

**Table 2:** Participants’ characteristics.

ID	Age/sex	Affected side	Poststroke period	BBS score
S1	51/male	Left	2 months	31
S2	47/male	Right	1.5 years	21
S3	57/male	Right	7 days	46
S4	70/male	Left	3 years	31
S5	58/male	Right	3 years	53
S6	36/male	Left	1 years	46
S7	60/male	Right	5 months	30
S8	56/male	Left	1 month	36
S9	74/male	Left	8 days	51
S10	52/male	Right	2.5 years	23
S11	57/male	Left	3 months	40
S12	45/female	Left	8 months	25

*BBS, Berg balance scale*.

### Experimental Setup

**Figure 7:**
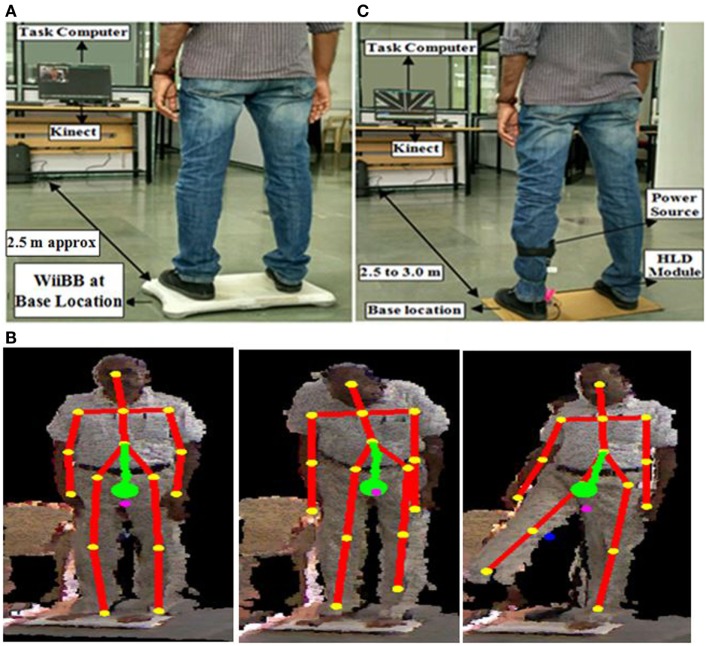
**(A)** Experimental setup. **(B)** Statistically equivalent serial chain identification stage. **(C)** Threshold estimation and task execution stages. WiiBB, Wii balance board.

We designed a usability study that used a Kinect, WiiBB, and task computer (PC). This study consisted of three stages, namely (i) SESC identification stage, (ii) threshold estimation stage (for individualized weight-shifting), and (iii) task execution stage. During the SESC Identification stage, the experimental setup consisted of Kinect, WiiBB, and the PC. The WiiBB was positioned at a Base Location on the ground (Figure 7A) that was approximately 2.5 meter in front of the PC. The Kinect sensor (for tracking the participant’s movement) was connected to the PC kept on a table top. In the Threshold Estimation and Task Execution stages, the experimental setup remained the same, except, the WiiBB was removed from the Base Location. An HLD module was attached to the participant’s affected leg (Figure 7C).

### Experimental Protocol

Our study required a commitment of approximately 30 min from each participant. Once the participant arrived, a physiotherapist in our team assessed the participant’s residual balance using Berg balance score (BBS) (Berg et al., 1992) and also ensured that the inclusion criteria (see Participants) were satisfied. Then, the experimenter showed the experimental setup to the participant and demonstrated the VR-based tasks. This was followed by the signing of consent forms.

Once the participant was ready, the experimenter started the experimental study that comprised of three stages (see Experimental Setup). First, the SESC Identification stage was carried out. In this, the participant was asked to stand upright on the WiiBB (Figure 7A) kept at the Base Location and facing the Kinect placed close to the task computer. Then the participant was asked to make a series of static postures, namely, lean forward and lift one leg at a time away from the body (Figure 7B). Once the SESC parameters were identified, we asked participants to repeat the same static body posture once again and recorded the measured CoP data from WiiBB and estimated CoM data using SESC method to compare the variation in CoP and CoM movement (*CoP-CoM comparison stage*). This stage was followed by the Threshold Estimation stage (see Experimental Setup). Before participating in this stage, the participant was asked to sit on a chair and wear a pair of shoes with the help of the experimenter, who in turn mounted the HLD module (see Monitoring of Ankle Strategy during Task Execution) on the shoe of the affected leg (Figure 4B). Also, the experimenter removed the WiiBB from the Base Location and asked the participant to stand on the ground at the Base Location (Figure 7C) while facing the Kinect sensor. Subsequently, the experimenter asked the participant to shift his weight in the *N* (forward), *E* (right side), and *W* (left side) directions to his maximum ability while following ankle strategy and not moving from the Base Location. Finally, the Task Execution stage was carried out in which the participant was asked to stand on the ground at the Base Location and interact with the VR-based balance tasks by shifting weight in different directions. The VR-based balance task (Figures 2 and 3) required them to shift their weight in the instructed direction to maneuver the virtual object (*VR_Obj_*) in the VR environment from *Central_Hold_* to *Target* position (see Design and Control of VR-based Balance Rehabilitation Tasks). At the end of the study, the participant was asked to respond to a questionnaire used by the experimenter to get the participant’s feedback on our Virtual CoMBaT system.

### Statistical Analysis

While the participants interacted with our VR-based tasks during the Task Execution stage, Virtual CoMBaT computed the participant’s performance (*P_S_^x^*; see Performance Evaluation Module) corresponding to each direction in the tasks offered in each difficulty level. We were interested to understand whether the repeated exposure to tasks in each difficulty level contributed to any statistical improvement in one’s performance. Before performing the statistical test of the hypothesis, we performed Shapiro–Wilk test of normality (Shapiro and Wilk, 1956) on the participant’s performance score in different directions of tasks of DL1 and DL2. For the sample size of 12, we computed W value and found with significance level of *p*-value = 0.05 that the data was not normally distributed Subsequently, we opted for non-parametric statistical hypothesis testing, namely, Wilcoxon signed rank test (Gibbons and Chakraborti, 2011) to determine whether the improvement (if any) in the participants’ performance score from their task in the *First Attempt* to task (within each difficulty level) in which they achieved Best score (*Best Case* henceforth) in each direction (such as. *N, W, E, NE*, and *NW*) was statistical. Thus, here we have used Wilcoxon signed rank test keeping the *First Attempt* and *Best Case* attempt as between-subject factor and DL1 and DL2 as within-subject factor. Also, for paired difference between performance scores obtained in different directions (*N, W, E, NE*, and *NW*), we have used a multiple comparison correction method such as Holm correction method (Aickin and Gensler, 1996). The test was performed with the significance level set at *p* < 0.05.

## Result

In the current work, we have designed a Virtual CoMBaT system. Our aim was to design a usability study to understand the users’ perspective on the usage of Virtual CoMBaT system. In the section below, we present our findings on the participants’ views on the usage of our system. Additionally, since, Virtual CoMBaT was designed with an ultimate aim to serve as a rehabilitation tool, we analyzed the data of our usability study to see whether this system can also contribute to at least some improvement in performance even over a limited exposure duration. Thus, when the participants interacted with the Virtual CoMBaT system, we monitored their weight-shift capability while they followed ankle strategy. To ensure that the ankle strategy was followed, we have designed HLD module. Here, we will present our observation on the implication of the HLD assisted Virtual CoMBaT system on the participants’ weight-shifting status along with improved usage of ankle strategy. In turn, we investigate whether the Virtual CoMBaT system (1) can offer a mechanism to quantify the balance ability of individuals based on one’s ability to shift weight in different directions and (2) with a personalized CoM can have implication on one’s performance in terms of improved weight-shifting capability. Here, we present the result of our usability study in which 12 stroke patients volunteered.

### Participants’ Feedback on System Usability Questionnaires

From the participants’ responses, we found that the participants did not face any difficulty in understanding the tasks (except S6 and S10) and were in fact interested in interacting with our system. Also, with one-day exposure to Virtual CoMBaT, they could realize the potential benefits that the system can bring to them as far as balance rehabilitation was concerned. Thus, they expressed their willingness to interact with our system again in future and also refer their known acquaintances to this study. Thus, from the participants’ feedback, we can infer that Virtual CoMBaT system has the potential to be accepted by individuals with balance disorder. Table 3 shows the average response scores of the participants.

**Table 3:** Participants’ feedback.

Q. no	Suitability evaluation questions	Average response score
Q1	Did you face any difficulty in understanding the tasks?	1
Q2	Did you find the tasks interesting?	4
Q3	Do you think you can benefit by using such a system?	4
Q4	Do you want to play again with this system?	5
Q5	Do you want to refer any of your acquaintance to our study?	5

*1 = strongly disagree, 2 = disagree, 3 = neutral, 4 = agree, and 5 = strongly agree*.

### Virtual CoMBaT System Used for Quantification of Balance Ability Based on Weight-Shifting Capability

**Figure 8:**
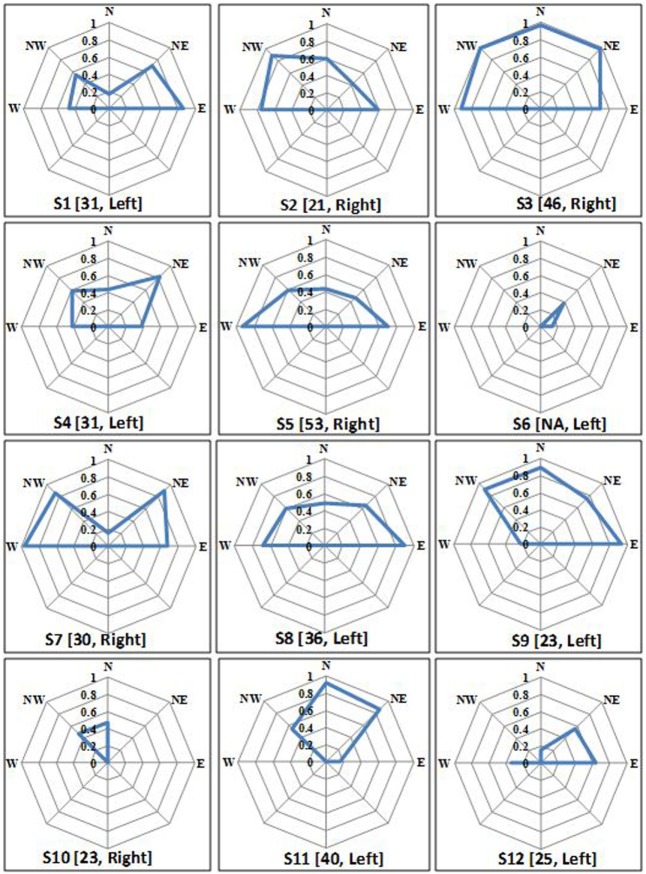
Individual normalized equivalent performance score in all five directions. Notation written below each plot = participant’s ID (Berg balance scale score, affected side).

We wanted to see whether Virtual CoMBaT system can quantify one’s ability to shift weight in the five different directions using the task performance measure. We analyzed one’s NEP (see Performance Evaluation Module) using estimated CoM. Figure 8 shows each participant’s *NEP* for all the five directions, their BBS scores, and hemiplegic side. From Figure 8, we see that the participants’ *NEP* was lesser in a direction(s) that required weight-shifting on their affected side for maneuvering the *VR_obj_* in that direction compared to the other directions. For example, participant S1 was left hemiplegic and thereby had restricted weight-shifting capability on the left side. This caused him to score less in the VR-based tasks while shifting his weight to maneuver the VR_obj_ toward the *W, NW*, and *N* directions than that in the other directions. Specifically, *W and NW* directions needed him to shift his body weight more toward his left side thereby transferring a major part of his body weight on the left leg (the weaker side). Again, maneuvering VR_obj_ toward *N* direction would ideally need equal contribution from both left and right legs to facilitate proper weight-shifting. But, since S1 was left hemiplegic, his restricted weight-shifting capability toward the left side of his body resulted in the VR_obj_ to be maneuvered more toward the *NE* direction than toward the *N* direction, thus scoring less for the *N* direction. The opposite was the case of weight-shifting for participant S2 who was right hemiplegic. There were some exceptions, such as, participant S3 who was right hemiplegic and can be expected to perform badly in the *E, NE*, and *N* directions. However, we find that S3 has performed well in all the directions except for the *E* direction. A possible explanation can be that S3 had most of his residual motor capabilities intact. His clinical report said that he had a very mild stroke and his high BBS score reflected that he was not suffering from severe balance disorders.

The weight-shifting profile (Figure 8) can provide a quantitative pictorial representation of direction-specific weight-shifting capability of an individual. This information on one’s direction-specific current balance status can aid a therapist to plan rehabilitation exercises in an individualized manner. Though we have tried to connect the direction-specific weight-shifting capability with the hemiplegic side, yet we do not want to generalize our findings given the limited participant pool.

Again, we wanted to understand if there existed any correlation of the quantitative clinical measure with one’s task performance while participating in the study with Virtual CoMBaT. As we had the participants’ BBS data, we wanted to use this data as the quantitative clinical score. Since, (i) our participants were all hemiplegic and had direction-specific performance score (*N*, *E, W, NE*, and *NW*) while participating in the usability study and (ii) BBS score is not a measure of one’s direction-specific weight shifting ability, we decided to choose that measure out of the 14 measures (each scaled 1–4) of BBS score that can provide some direction-specific information. Specifically, we chose the score for “Reaching Forward with Outstretched Arm while Standing” task of BBS (when the participant needs to shift weight toward the front (*North*)) and tried to understand the correlation of this quantitative clinical score with participants’ task performance score toward the *North* direction (that is when the participant shifted weight toward *N* direction) in the *First Attempt* of DL1 task. For computation of the correlation, we considered the score for 8 (S1, S2, S4, S5, S7-S9 and S12) out of the 12 participants, since, only for these participants we had BBS scores for each of the 14 measures of BBS and only the total score (without breakup) for the remaining. The correlation was found to be 0.84.

### Effect of Interaction with Virtual CoMBaT System on one’s Performance

**Figure 9:**
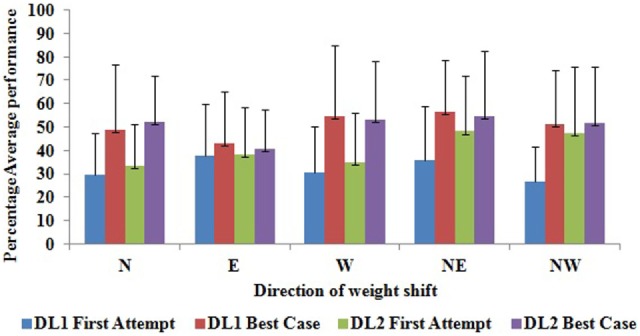
Group average of % performance score in *First Attempt* and *Best Case* attempt in each direction. DL1, difficulty level 1; DL2, difficulty level 2.

While we designed the usability study of Virtual CoMBaT system, we also wanted to understand its potential to improve one’s performance in terms of improved weight-shifting, since this system was designed with an ultimate aim in mind that this will be used as a rehabilitation tool. So, we wanted to see whether there was any scope of improvement even during its usage for a limited duration that is one day. Then only we might be able to judge its worthiness to be used over extended period for rehabilitation. To understand the potential of Virtual CoMBaT to contribute to the improvement in one’s weight-shifting capability across the VR-based tasks, we computed one’s performance in the *First Attempt* and the *Best Case* attempt achieved in tasks belonging to each difficulty level (DL1 and DL2). Figure 9 shows the comparative representation of the group average of the participants’ performance in all directions for *First Attempt* and *Best Case*. The group average (irrespective of the hemiplegic side) shows improvement (Δ) in the % performance score from the *First Attempt* to *Best Case* (Δ = 61.85% for DL1 and 24.1% for DL2) averaged over all the five directions.

Again, the *First Attempt* and *Best Case* attempt were separated by intermediate trials and its number varied across participants.

From Table 4, we can see that for all participants (except S2, S6, and S10) the number of trails executed before reaching the *Best Case* attempt were more in DL2 than that in DL1, as expected, since, DL2 was comparatively more challenging than DL1. A possible reason behind S2 having lesser number of trials in DL2 than DL1 before reaching the *Best Case* attempt might be that S2 having the least BBS score was considerably challenged in his balance ability. Consequently, he took most of the time from the 20 min exercise window in interacting with DL1 tasks before moving to DL2, unlike other participants. For S6 and S10, we find that both of them took the entire 20 min of exercise time in interacting with DL1 tasks. A possible reason behind this can be that both of them were having issues in understanding the tasks and following the experimenter’s instructions that took them long to finish each task, as reported by the experimenter. Also, we observed a group variability of approximately 10% and 8% in performance scores across DL1 and DL2, respectively, on an average in the trails executed before reaching the *Best Case* attempt. Additionally, we could find an overall improving trend in the % performance score across the trials while going from the *First Attempt* to *Best Case* attempt for most of the participants.

**Table 4:** Number of trials needed to reach *Best Case* attempt.

Participants’ ID	Number of trials	
	DL1 (no.)	DL2 (no.)
S1	3	18
S2	9	2
S3	2	18
S4	5	7
S5	9	13
S6	4	NA
S7	2	22
S8	2	13
S9	1	17
S10	8	NA
S11	5	11
S12	3	5

*The number of trials is inclusive of *First Attempt* in both DL1 and DL2. NA indicates not applicable*.

Table 4 indicates the number of trials (inclusive of the *First Attempt*) before reaching the *Best Case* for each participant. For the DL1 tasks, with respect to participant S9, the Table 4 indicates the number of trials needed to reach the *Best Case* was only 1. This number includes the trial for the *First Attempt*. Specifically, S9 interacted with two trials in DL1, i.e., one trial for the *First Attempt* that happened to be the *Best Case* for him and one trial in which he scored less than the *First Attempt* before the task switching engine (see Task Switching Module) switched him over to the DL2. Results of our data analysis indicate that the percentage change (%Δ) in performance score from the *Best Case* attempt to that in the next trial before switching over to the DL2 was approximately 3%. Again, with respect to participants S1, S7, S8, and S12, though the number of trials (inclusive of the *First Attempt* in DL1) needed to reach the *Best Case* was only 3, 2, 2, 3 trials, respectively, yet, the corresponding%Δ in the performance score was approx. 23, 11, 2, and 3%, respectively. For the participant S3, the%Δ in performance score from the *Best Case* attempt to that in the next trial before switching over to the DL2 was very less (approx. 0.2%). However, S3 scored approx. 92% in all three trials (*First Attempt, Best Case* attempt and the one before switching over to the DL2) and thus the fourth task trial (first trial in DL2) contributed by helping to break the monotony. However, further fine tuning of the Task Switching rationale with modification of Condition 1 (Table 1) is possible by using a specific value of%Δ in performance score (from the *Best Case* attempt to that in the next trial before switching over to the DL2), say *x*% change, with *x* = 2% (say, as a typical case) for switching to tasks of higher difficulty level.

Before performing the statistical test of the hypothesis, we performed Shapiro–Wilk test of normality. From the W statistics, we found that for most of the directions, the average performance score (%) was not normally distributed particularly for *First Attempt* of both DL1 and DL2 tasks with a significance level of *p*-value = 0.05. Thus, while computing the improvement in the average performance score (%) of participants from *First Attempt* to the *Best Case* attempt, a dependent sample Wilcoxon signed rank test was carried out on the score in tasks belonging to DL1 and DL2. Also, we performed multiple comparison corrections using Holms method on the *p*-values obtained for improvement in performance score for different directions (*N, E, W, NE*, and *NW*). From the results, we observed a statistically significant improvement (*p*-value = 0.024 for *N*, *p*-value = 0.015 for *W*, *p*-value = 0.016 for *NE*, and *p*-value = 0.042 for *NW*) in the% performance score from *First Attempt* to *Best Case* for the different directions (except for direction *E*). A possible reason for the improvement in performance score along *East* direction being non-statistical can be that 7 out of the 12 participants (Table 2) were left hemiplegic who performed better while shifting weight toward *East* direction throughout the balance task than that toward other directions irrespective of the task trial. However, for DL2, the improvement in the performance score was not significant. A possible reason for this could be that the DL2 tasks being of highest difficulty level required more weight-shifting to improve the performance score considerably. Further investigations over extended study duration with more participants are warranted before generalizing such observations.

### Effect of Virtual CoMBaT System on Following of Ankle Strategy

**Figure 10:**
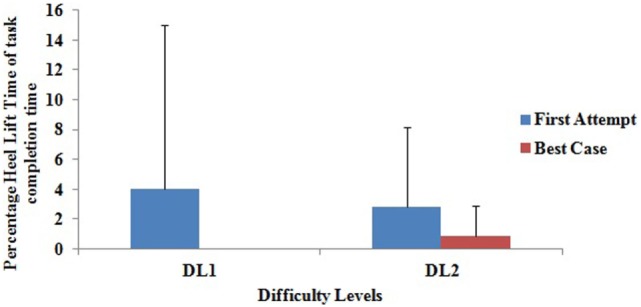
Group average of % heel lift duration.

While interacting with the Virtual CoMBaT system, we encouraged the participants to follow ankle strategy. Our HLD module (see Monitoring of Ankle Strategy during Task Execution) was used to record the participant’s heel lift during Task Execution stage. While measuring one’s heel lift, we recorded the duration for which a participant lifted his/her heel (*Not Following* ankle strategy). Subsequently, for each task trial, we computed the duration of one’s heel lift (if any) as a percentage of the total time taken to execute that task trial. Here, we present our findings on the% heel lift time (out of the total time taken) for each of the *First Attempt* and *Best Case* tasks for both DL1 and DL2 tasks. The aim was to understand whether repeated exposure to our Virtual CoMBaT system facilitated the participants to acquire improved weight-shifting capability while reducing the duration of heel lift. Figure 10, shows that there was a reduction in the average group percentage of time (out of total task trial duration) the participants had lifted their heels from the ground surface from their *First Attempt* to *Best Case* while executing tasks in each difficulty level. We can see that for DL1, during the *First Attempt*, they were frequently lifting their heels while shifting weight. In contrast, in their respective *Best Cases*, none lifted their heels while performing their task trials in DL1. For the more difficult tasks, namely, DL2, the participants lifted their heels in their *First Attempt* as well as during their respective *Best Cases*, but the amount of time of heel lift in the *Best Case* was 68.92% less than that for the *First Attempt*. Thus, the reduction in heel lift time from the *First Attempt* to the *Best Case* indicates improvement in terms of better adherence to ankle strategy during weight-shifting by the participants.

## Discussion and Limitation

In our present study, we designed an individualized Virtual CoMBaT system for balance rehabilitation that required the participants to perform the VR-based weight-shifting tasks while using ankle strategy. The main contribution of our present work is the design of CoM-assisted Balance Training platform having a VR-based system augmented with Kinect to offer balance rehabilitation exercises in an individualized and adaptive manner based on one’s performance capabilities. Additionally, to facilitate the participant in adhering to the usage of ankle strategy during weight-shifting, we developed US-based shoes that can sensitize a participant with audio alarm if he/she deviates from following the ankle strategy. Finally, to test our system, we carried out a usability study to understand the implications of such a system on one’s balance.

A usability study was carried out with 12 stroke survivors. From participants’ feedback, we could find that our system has a potential to be accepted by the target population. Additionally, since we designed Virtual CoMBaT system with an ultimate aim that it can be used as a rehabilitation platform, we wanted to understand whether it has a potential to contribute to improvement in one’s weight shifting ability even with a limited exposure. Results indicate the potential of our system to contribute to improving one’s overall performance in tasks belonging to different difficulty levels. In fact, with our study being carried out for a limited duration (one day here), we could see statistical improvement in performance score from *First Attempt* to the attempt in which they attained Best performance for the tasks of DL1. But, we could not see any statistical improvement in performance in tasks of DL2 that were more difficult than tasks of DL1. Again, improvement in task performance necessitated a participant to be able to physically do increased weight shifting along with improved adherence to ankle strategy, all of which might indicate improved rehabilitation effect instead of rote learning effect. Since our participants were hemiplegic, we wanted to go further in data analysis to see whether such a system can contribute to improvement in one’s capability to shift weight in specific directions, crucial for quantification of patients’ asymmetric balance in different directions and also a critical component of performing daily-living tasks. Thus, we computed one’s NEP as a balance-related metric associated with one’s direction-specific weight-shifting capability. Additionally, results indicate that our Virtual CoMBaT system can also provide quantitative measures that can help one to quantify the participant’s initial ability in shifting weight in different directions.

Though the results are promising, yet, our study had some limitations such as, our task switching engine offered a different task to a participant (who had already scored Adequately) from DL1 to DL2 as soon as his performance in the current task decreased from his preceding task (Condition 1 in Table 1) that might make our task switching rationale sensitive to small fluctuation (that is, decrement) in % performance score. Although in this study, for all the participants we could see a minimum of 2% change from the *Best Case* attempt to that in the next trial before switching over to a task of DL2 (except for S3), we could have fine tuned the system to switch tasks whenever the% change in performance score was beyond a limiting value, say *x*%, with value of *x* determined based on the study conditions. In future, we plan to fine tune the task switching rationale with modification of Condition 1 (Table 1) which might improve the capability of our Virtual CoMBaT system as far as the balance training is concerned. Other limitations of the current study were small participant pool, patients with varied poststroke period and different hemiplegic sides, and limited duration of exposure to the system. This study was used to administer exercises among participants only for one session of balance training. Such a limited exposure may not be sufficient to speak on the rehabilitation efficacy of the system. To see a significant improvement in one’s clinical measure of balance, one needs to carry out a longitudinal study. Also, this must be associated with clinical assessment of balance ability by measuring BBS score prior to and post the study. In future, we plan to carry out a more in-depth longitudinal study incorporating larger participant pool before such a platform can be deployed in clinical settings. For our present study, we recruited participants having a widely varying poststroke period and different residual balance capability based on the availability. This might have affected the group average of the participants’ performance scores. In future, we plan to extend this study by enrolling a larger patient population categorized based on residual balance capability before exposing them to our Virtual CoMBaT. This would enable us to carry out in-depth statistical analysis to get better understanding on the statistical variations in the balance-related clinical measures upon exposure to our Virtual CoMBaT system. Another limitation of our system is with estimation of thresholds in the computation of one’s performance score in a task. Specifically, for the sake of simplicity and lack of available database on stroke patients, we tried to decide threshold measure such as, *D*_TH_ in Eq. 4 based on pilot trials with age-matched healthy participants. The choice of such thresholds might have implications on the performance score of the stroke group for whom the thresholds might be different. However, this was an initial approximation. In future, we plan to extend our study while modifying parameters such as, threshold measures while computing the performance scores by using the database that we have developed in our present study with poststroke patients.

Given the potential of our Virtual CoMBaT to contribute to improved balance-related performance of patients with balance disorders, this system deployed in the usability study can be extended to be used as an alternative personalized rehabilitation platform at clinics and home-based settings. Though through our usability study, we could understand the potential of Virtual CoMBaT to contribute to balance rehabilitation, yet this study was carried out in a controlled environment. Thus, questions remain on the transferability of the skills learned from the controlled environment to real-life situations.

## Ethics Statement

The study was conducted by following the institutional research ethics. All participants provided informed consent for their participation in the study.

## Author Contributions

DK and UL drafted the manuscript and contributed to the experiment design, experimental data collection with stroke participants, data analysis, and statistical analysis. AG, MH, and PF contributed in developing the algorithm for estimation of body center of mass. AD contributed to research by giving clinical advice. Both AD and AD contributed to developing the procedure for data collection. All the authors read, corrected/commented, and approved the final manuscript.

## Conflict of Interest Statement

The authors declare that the research was conducted in the absence of any commercial or financial relationships that could be construed as a potential conflict of interest.
